# Development of a Rapid Method for the Determination of Caffeine in Coffee Grains by GC-FID—A Fully Validated Approach

**DOI:** 10.3390/antiox6030067

**Published:** 2017-08-22

**Authors:** Ioannis N. Pasias, I. Kiriakou, Charalampos Proestos

**Affiliations:** 1 General Chemical Lab of Research and Analysis, Timfristou 181, 35100 Lamia, Greece; iopas@chem.uoa.gr; 2 Lamia Laboratory, Karaiskaki 85, 35100 Lamia, Greece; info@lamialab.com; 3 Laboratory of Food Chemistry, Department of Chemistry, National and Kapodistrian University of Athens Panepistimiopolis Zografou, 15771 Athens, Greece

**Keywords:** caffeine, uncertainty calculation, ISO 17025, Guatemala coffee grains, GC-FID

## Abstract

A simple method for the determination of caffeine in coffee grains by GC-FID (Gas Chromatography-Flame Ionisation Detector) is presented in the current work. The method was fully validated according to ISO (International Organization for Standardization) 17025 requirements and European Commission regulations. The accuracy, as provided by recovery experiments, was higher than 93%, and the precision, as provided by the (%) relative standard deviation under reproducibility conditions, was lower than 5%. A vast number of independent parameters that lead in the increase of uncertainty of methods were investigated. The analysis was performed without use of an internal standard, which was proven to be reliable according to several validation methods. The method was applied in real samples, and possible health claims were investigated.

## 1. Introduction

Current food industry trends include the production of new functional foods, so far known as “superfoods”, with pro-health properties through the introduction of components with antioxidant or antimicrobial activity [1]. Recently, green coffee has been introduced as a food supplement, regarding its functional and antioxidant activity, since it contains phenolic compounds and caffeine [1]. On the other hand, caffeine is a stimulant, which has different health effects concerning stimulation of the central nervous system and can produce restlessness, headaches, and irritability [2]. Large amounts of caffeine consumption can cause physiological and psychiatric dependence [2]. The European Food Safety Authority mandates that energy drinks with over 150 parts per million (ppm) or 150 mg/L caffeine content should be labeled as having “high caffeine content” and the exact amount should be indicated; this rule excludes tea, coffee, and cocoa [3].

Many different modern analytical methods are proposed in the literature for the determination of caffeine in coffee samples. All these different methods are perfectly presented in an excellent review by Jeszka-Skowron et al. (2015) [4]. Caffeine is usually determined in coffee samples by the use of liquid chromatography combined with different detectors, such as UV or mass spectrometers, and rarely by gas chromatography [4,5]. Sample preparation before this analysis is usually very simple and contains only hot water extraction, filtration, and dilution steps. However, liquid chromatography sometimes demands the use of complicated mobile phases, as well as careful pH adjustment, procedures which are both time- and cost-consuming [4].

The aim of the current study was to develop a rapid, accurate, precise, and low-cost analysis for the determination of caffeine in coffee grains by GC-FID without using an internal standard. For this reason, special precautions for the accuracy and the precision of the method were taken and presented for the first time. Finally, a whole validation protocol guide according to the ISO 17025 accreditation standard is presented [6].

## 2. Materials and Methods

### 2.1. Instrumentation

All measurements were performed with a Shimadzu GC 2010 PLUS, GC-FID system, using an Agilent DB-1 (30 m × 0.32 mm × 1 μm) column. The optimized conditions were: injection volume, 1 μL in split mode 50:1; injector temperature, 280 °C; carrier gas, He at a constant flow of 1.8 mL/min; and detector temperature, 300 °C. The initial oven temperature, 100 °C, was held for 2 min, then programmed to increase at 8 °C/min to 180 °C, and finally programmed to increase at 10 °C/min to 250 °C, where it was held for 0 min.

### 2.2. Analytical Procedure

Three different qualities of coffee grains were purchased from the local markets in Lamia, Greece. From each category, three different samples under the same brand name were purchased in order to estimate the average content of each sample. Samples were homogenized in a mortar.

Then, 0.5000 g of the homogenized coffee grains were extracted with 10 mL of ultra-pure water and by heating for 15 min at 100 °C. The samples were then back-extracted to 10 mL dichloromethane and the 1 μL of extract was injected into the GC system. For method accuracy, the same procedure was followed for fortified samples spiked with a known amount of analyte at three different content levels, and the estimation of recovery and % relative standard deviation under inter- and intra-day precision was performed. In order to avoid the use of an appropriate internal standard, a quality control chart was constructed and the internal quality sample was analyzed after five different runs.

### 2.3. Method Validation

The method was fully validated in view of the Commission Decision of 12 August 2002, implementing Council Directive 96/23/EC concerning the performance of analytical methods and the interpretation of results [7].

Both standard addition and external calibration curves were constructed in order to check possible matrix interferences by comparing the slopes using the *t*-test as a criterion. The instrumental and methods limit of detection was calculated by multiplying the standard error of the intercept by 3.3 and dividing the calculated result by the slope. A similar procedure was followed for the calculation of the limit of quantification, but the factor for the intercept multiplication was equal to 10.

The intra- and inter-day precision under repeatability and reproducibility conditions, respectively, were also estimated. In Thompson’s work (2000), the precision performance criteria were perfectly presented [8]. As the method’s precision performance criteria, Thompson (2000) used the HORRAT_r_, meaning the observed relative standard deviation (% RSDr) under repeatability conditions divided by the RSDr value estimated from the Horwitz equation, using the assumption that r = 0.66R, as well as the HORRAT_R_ values, meaning the observed RSD_R_ value under reproducibility divided by the RSD_R_ value calculated from the Horwitz equation. Thompson (2000) concluded that, for a precise analysis, both values should be less than 2 [8].

The accuracy of the method was estimated by the calculation of the % recovery for three different fortified content levels, each measured six times. Recovery data were considered as acceptable when they were within ±20% of the theoretical fortified content [7].

Internal quality control charts (IQCs) were also constructed in order to monitor whether results were reliable enough to be released. The objective of IQCs is the elongation of method validation, achieved by continuously checking the accuracy of analytical data obtained from day to day in the laboratory. The analytical system is under control if no more than 5% of the measured values exceed the warning limits and none of them exceed the action or control limits [9,10,11,12,13]. The quality control chart was constructed by an internal quality fortified sample of coffee measured after five runs.

The uncertainty of the method was also calculated based on the Eurachem/Citac Guidelines [14]. In practice, the uncertainty of the results in this study arose from many possible sources, including matrix effects and interferences, environmental conditions, uncertainties of masses and volumetric equipment, stock standard solution reference values, approximations and assumptions incorporated in the measurement method and procedure, and random variation. The combined uncertainty uc (y) was calculated from the summary squared of several independent parameters such as (a) the mass uncertainty; (b) the dilution volume uncertainty; (c) the calibration uncertainty; (d) the bias uncertainty, as estimated by the recovery tests; and (e) the precision uncertainty, as estimated by the % RSD_R_ values for the three different concentration levels under reproducibility conditions. The choice of the factor k is based on the level of confidence desired. For an approximate level of confidence of 95%, k is 2.

## 3. Results and Discussion

### 3.1. Results of Method Validation

**Figure 1:**
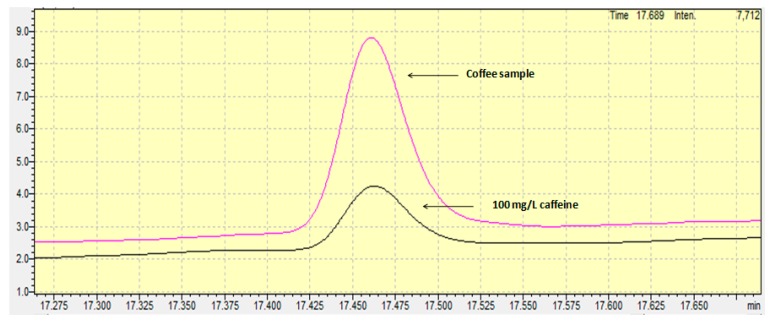
Chromatograms of caffeine in a standard solution and in a coffee grain sample.

Standard addition calibration and external calibration curves were used as quantification techniques. Internal standard solution was not used in the current work. In general, the use of an internal standard in the GC-FID analysis is commonly used to correct random errors from the injection repeatability and systematic errors from the instrumental drift and the procedural errors. However, as defined in the review by Hewavitharana (2009), the internal standard can become a foe “if the analyst is unaware of the linear range of the internal standard and uses a concentration that is outside the linear range, a severe loss of accuracy will occur” [15]. Furthermore, the appropriate use of the internal standard suggests that all samples must be spiked with the appropriate content of the internal standard during the first step of the preparation step, meaning the sample weighing. In this step, usually few microliters are spiked in some grams, and for this reason the accuracy of the method depends on the homogenization of the sample mixture. The whole procedure is time- and cost-consuming and for this reason, in the current work, no internal standard was used with regard to special precautions. Concerning the quantification technique, external calibration was finally chosen, since the *t*-test proved that no significant difference existed between the two slopes (the external standard calibration curve and the standard addition calibration curve). The linear range of the calibration curve spanned from 10 to 1000 mg/L, a range that is “fit for purpose” for avoiding time-consuming dilutions of the samples and for having the centroid standard solution of the calibration curve close to the content of the samples, in order to achieve more accurate results. Figure 1 shows the chromatogram achieved from a standard solution containing 100 mg/L of caffeine.

The limit of detection, as provided from the standard error of the intercept of the calibration curve, was found to be equal to 3.1 mg/L or 62 mg caffeine/kg. The limit of quantification was found to be equal to 9.3 mg/L or 186 mg caffeine/kg. Comparing the limits of detection and quantification with those achieved from other works and by other methods, it can be concluded that the values determined in the current work are similar to or slightly worse than others, but they are surely “fit for purpose” regarding the content levels of caffeine in coffee grains. For example, a better limit of detection was achieved compared to the HPLC-UV method proposed by Fernández et al. (2000), and a similar value was determined compared to that achieved by derivative-spectrophotometry as proposed by Alpdogan et al. (2002) [16,17]. On the other hand, the limits of detection were worse than those proposed by Shrivas and Wu (2007), Song (Sherry) and Ashley (1998), and slightly worse than that proposed by Frizzarin et al. (2016), who determined the levels of caffeine using a fully-automated in-syringe dispersive liquid-liquid microextraction [18,19,20].

The precision was estimated by spiking the coffee samples with 0.5 (%w/w), 1.0 (%w/w), and 2.0 (%w/w) caffeine, with respect to the background content of the coffee samples. Each fortification level was measured in triplicate in the same day (intra-day precision) and on two different days (inter-day precision). The results proved that, even though no internal standard solution was used, the % RSDr (intra-day precision) and % RSD_R_ (inter-day precision) for all fortification levels were lower than 5%, and the HORRATr and HORRAT_R_ values were lower than the crucial value of 2 (Table 1). The results proved that, for high content levels, the uncertainty of the volume injection reading does not have an effect on the results precision with or without using an internal standard.

The accuracy of the method, as calculated by the recovery of the three different fortification levels, was found to be higher than 93.5% (Table 2). The method was considered “fit for purpose”. The spiked sample fortified with 1.0% (w/w) caffeine was defined as the internal quality control sample used for the construction of the internal quality control chart. After five different consecutive runs, the internal control sample was analyzed and the value was recorded in the internal quality control sample. It was observed that in whole analysis procedure, there was no trend for the measured values to exceed the warning limits or the action limits, and therefore the analytical system was considered as “under control”.

The uncertainty of the method was calculated for all different fortification levels. The mass and volume uncertainty did not seem to significantly affect the expanded uncertainty. The most significant parameters were the calibration uncertainty, the bias uncertainty, and the precision uncertainty. The calculated expanded uncertainty was found to be equal to 16.0%, 14.5%, and 14.0% for the 0.5 (%w/w), 1.0 (%w/w), and 2.0 (%w/w) fortification levels, respectively. 

### 3.2. Health Claim

The developed and fully validated method was applied for the determination of caffeine in different Guatemala coffee grain samples (*n* = 3) (Figure 1). The mean caffeine content was found to be equal to 1.3% (w/w). Taking into account that a cup of coffee contains 80 mg of coffee, the mean intake content of caffeine for an adult is equal to 1.04 mg per day. This means that the weekly caffeine intake for an adult is equal to 7.3 mg. In Richards and Smith’s (2015) excellent work, the association between caffeine and different psychological situations were investigated. According to their work, the weekly intake of 7.3 mg found in the current work may cause anxiety in males and stress and depression in both males and females [2]. However, it is possible that the effects observed in the work by Richards and Smith (2015) are attributable to personality characteristics associated with caffeine users, rather than to their use of caffeine [2]. Future research should therefore aim to conduct intervention studies in order to investigate the nature of these relationships further.

## 4. Conclusions

In the current work a rapid, precise, accurate, and low-cost method for the determination of caffeine in coffee grains was developed without using an internal standard. The method was considered as “fit for purpose” according to the requirement of ISO 17025, and European regulation concerning the aspects for the accurate development of analytical methods. The recovery of the method ranged between 93.5% and 102%, and the % relative standard deviation was lower than 5%. The RSD bias and the inter-day precision provided higher uncertainty in the method. The Guatemala coffee grains analyzed by the developed method contain a high amount of caffeine, and can be used as functional food but may also lead to depression and anxiety.

## Figures and Tables

**Table 1:** Results of intra- and inter-day precision in three different fortification levels.

Fortification Level	% RSDr	% RSD_R_
0.5	4.1	4.5
1.0	2.5	3.4
2.0	2.0	2.2

**Table 2:** Results of accuracy in three different fortification levels.

Fortification Level	% Recovery	Standard Deviation
0.5	93.5	4.3
1.0	96.7	3.3
2.0	102.0	2.2
